# Substantial variation among Medicare beneficiaries in the impact from 2025 Part D out of pocket spending caps

**DOI:** 10.1093/haschl/qxaf059

**Published:** 2025-03-24

**Authors:** Debra M Lederman, Alexander L Olssen, Mark V Pauly

**Affiliations:** Health Care Management, University of Pennsylvania, Philadelphia, PA 19104, United States; Health Care Management, University of Pennsylvania, Philadelphia, PA 19104, United States; Health Care Management, University of Pennsylvania, Philadelphia, PA 19104, United States

**Keywords:** private retiree group insurance and other third party payments, out of pocket spending

## Abstract

In 2025, Medicare Part D introduced an annual $2000 limit on beneficiary out-of-pocket (OOP) costs under the Inflation Reduction Act. The objective of this research is to analyze the variability of OOP costs for beneficiaries with high total drug spending to understand who would benefit from the OOP cap. Using Part D data from 2022, we examine OOP costs for 2 samples of beneficiaries with high total drug spending: those with annual drug spending exceeding $6560 (who would have more than $2000 in OOP under the standard-benefit) and those in the top 1% of annual drug spending. We find that 64.72% and 37.84% of beneficiaries in each respective sample did not have OOP costs that would reach the annual cap. There is large variation in OOP costs even among beneficiaries who all have very high annual total drug spending, and reductions in patient liability from third-party payors appears to be an important reason for this variation. The introduction of OOP drug spending caps will result in substantial variation in the change in OOP spending for high-spending Medicare beneficiaries.

## Introduction

One of the most important features of the Inflation Reduction Act,^1^ to become effective in 2025, is an across-the-board annual limit on beneficiary out-of-pocket (OOP) spending of $2000.^2^ This increase in coverage over the original Part D insurance design will help beneficiaries by lowering the average OOP payment for non-poor (non “low-income subsidized”) beneficiaries. One underappreciated aspect of this policy is that, even among beneficiaries with similar (and high) levels of total drug spending, there will be large differences in how much benefit each beneficiary receives, with some benefiting not at all, while others will receive large cost reductions. Hence, many beneficiaries will not experience an increase in access and use of drugs, nor any cost offsets. This fact appears to be overlooked in current discussion of the policy, including in a simulation model developed by ASPE^3^ for the full set of IRA changes (including limits on spending growth), which shows about 5% of non-LIS beneficiaries will see a cut in OOP liability of $1000 or more in 2025. In this article, we focus on beneficiaries with high total drug spending (as opposed to those with high projected savings) and find that many beneficiaries, even those near the top of the distribution of drug spending, may see benefits that are small or even 0. While many have OOP liabilities for drug therapies with costs in the thousands of dollars, a significant minority only had to pay a few hundred dollars. This highly variable pattern of potential impact may affect both access to high-cost drugs and financing of Part D benefits.

Of course, capping what is paid OOP does not in itself save any Medicare cost or spending; the cap may increase Medicare's costs by reducing patient's costs and inducing utilization. However, the cap certainly transfers financial responsibility from some beneficiaries to higher premiums to be paid by all enrollees or taxpayers—this transfer is advantageous because it reduces financial risk (and potential future medical debt) for those who would have been subject to high OOP liabilities under the previous Part D design.

There are several features of the Part D market that sever the link between an individual's OOP spending and their total drug spending. As a result, the effect of the cap will likely differ even among those with similarly high total spending.

There are 3 major sources of variation in the impact of the cap on individual beneficiaries. First, most beneficiaries will be unaffected by the cap because they have modest annual prescription drug costs. Second, there is considerable variation across Part D plans in formularies, and OOP provisions, which affects how much beneficiaries will benefit from the cap.^4^ Part D beneficiaries receive coverage from standalone plans (PDPs) or Medicare Advantage Plans with drug coverage (MAPDs). Premiums for PDPs vary by a factor of 8, and MAPD plans often have 0 explicit premiums. A major source of OOP variation comes from coverage of the deductible, which is often reduced below the standard level or even eliminated entirely. Some people choose plans that meet the minimum actuarial value but impose cost sharing largely through per unit fixed dollar copayments linked to tiers of a formulary rather than coinsurance. Other provisions of cost sharing also vary, as does coverage of drugs. The third, not well-recognized source of variation arises when a beneficiary can access retiree group insurance^5^ that includes drug coverage. This privately financed coverage can “wrap around” benchmark Medicare-funded coverage (which is the primary payer). It is common among government employees (federal and other), universities, and other large employers. This additional coverage that reduces patient liability can take many forms depending on the plan chosen or offered, but frequently it has taken the form of a cap on OOP payment (at $2000 or less), even before the IRA provisions prevailed.

In addition, there has been extensive discussion^6^ of the evidence that Medicare beneficiaries do not always choose the Part D plan that minimizes their OOP cost, given their expected drug claims and premium. The flipside of this is that there must be variation in OOP payments across beneficiaries with similar (high) drug expenditure; some who make wise choices will pay much less OOP.

## Our analysis

We analyze 2 subsamples relevant to the impact of the IRA cap. Our first sample, presented in Table 1, includes all persons with total drug spending at $6560, greater than the minimum amount that would trigger OOP of $2000 under the standard plan. Our second sample, presented in Table 2, consists of beneficiaries with total spending in the top 1% of total spending. Our focus is on the extent to which OOP varies with total spending and, by extension, how OOP varies for a given high level of total spending. Throughout our analysis total drug spending is measured using the Part D total cost variable, which measures the price paid at the point of sale and does not account for manufacturer rebates or pharmacy direct or indirect remuneration. Importantly, coinsurance OOP is calculated from this price. All data is from a 5% sample of the Part D Event File provided by the Centers for Medicare & Medicaid Services (CMS).

**Table 1.** Average and median spending, by out-of-pocket (OOP) quartile for non-LIS beneficiaries with total spending exceeding $6560 and standard-benefit OOP >$2000.

	First	Second	Third	Fourth	
	OOP	OOP	OOP	OOP	Average
Spending	Spending	Spending	Spending	Spending	Across
Category	Quartile	Quartile	Quartile	Quartile	Quartiles
Average OOP	446.71	1211.93	1941.93	3785.42	**1846.50**
Spending ($)					
Median OOP	476.77	1208.03	1925.01	3028.89	**1659.68**
Spending ($)					
Average total	18 319.05	12 143.40	10 935.30	31 300.50	**18 174.56**
Spending ($)					
Median total	9977.42	7809.37	8793.35	16 875.80	**10 863.99**
Spending ($)					
Percentage with	84.11	45.74	23.45	14.92	**42.06**
Positive PLRO (%)					
Percentage with	100.00	100.00	58.87	0.00	**64.72**
OOP spending					
Below $2000 (%)					

Figures are based on 227 557 non-LIS beneficiaries, drawn from a 5% sample of Medicare beneficiaries for the year 2022, sourced from the CMS. The first out-of-pocket spending quartile consists of 56 890 beneficiaries. The second, third, and fourth out-of-pocket quartiles consist of 56 889 beneficiaries. Beneficiaries in the highest tier of total spending, specifically those in the top 1% of non-LIS beneficiaries with total spending exceeding $6560, have been excluded to eliminate potential outliers. The bolded values are the average amounts of all quartiles for each row.

**Table 2.** Out-of-Pocket (OOP), patient liability reduction (PLRO), and total spending metrics, by OOP quartile for non-LIS beneficiaries in the top 1% of total spending.

	First	Second	Third	Fourth	
	OOP	OOP	OOP	OOP	Average
Spending	Spending	Spending	Spending	Spending	Across
Category	Quartile	Quartile	Quartile	Quartile	Quartiles
Average OOP	350.48	2650.20	6496.65	11 676.23	**5293.39**
Spending ($)					
Median OOP	350.10	1898.20	6434.17	11 152.79	**4958.82**
Spending ($)					
Average total	99 470.28	87 508.56	81 059.89	180 528.37	**112 141.78**
Spending ($)					
Median total	78 387.79	63 207.60	79 215.52	173 249.23	**98 515.04**
Spending ($)					
Average PLRO	29 530.32	12 824.46	321.46	238.70	**10 728.74**
Spending ($)					
PLRO to	8425.68	483.91	4.95	2.04	**202.68**
OOP ratio (%)					
PLRO to total	29.69	14.66	0.40	0.13	**9.57**
Spending ratio (%)					
Percentage with	84.96	64.90	9.64	9.88	**42.35**
Positive PLRO (%)					
Percentage with	100.00	51.34	0.00	0.00	**37.84**
OOP spending					
Below $2000 (%)					

Figures are based on 18 588 non-LIS beneficiaries, drawn from a 5% sample of Medicare beneficiaries for the year 2022, sourced from the CMS. Each out-of-pocket spending quartile consists of 4647 beneficiaries. Beneficiaries in the highest tier of total spending, specifically those in the top 1% of the top 1%, have been excluded to eliminate potential outliers. The bolded values are the average amounts of all quartiles for each row.

In 2022, the standard Part D benefit included a deductible of $480. Copayment and coinsurance above the deductible were assumed to take the form of a standard 25% coinsurance. Under these policy provisions, a beneficiary would have hit a $2000 cap on OOP if their total spending exceeded $6560. This is calculated as follows: $480 (deductible) + 0.25 × $6080 (the amount spent above the deductible).

For the sample of beneficiaries in Table 1, who had spending above $6560, high enough to hit a $2000 OOP cap under the standard benefit, we observed that both mean and median OOP spending increased across quartiles of total spending. Mean OOP ranged from $446 in the lowest quartile to $3785 in the highest quartile of OOP spending, a factor of 9. Meanwhile, average total spending increased by less than double, rising from $18 319 to $31 300. This indicates that the proportion of financial responsibility borne by beneficiaries varied substantially within this set of high total spending beneficiaries.

A similar pattern is observed in Table 2 for the 1% sample of very high total spenders, which included everyone with total spending exceeding $45 373 and a median total spending of $89 051. The average OOP in the lowest quartile was much lower (at $350) than in the highest spending quartile (at $11 676). The proportion of total spending that beneficiaries paid OOP was considerably smaller for those in the lowest quartile, at 0.35% (350/99 470), compared with 6.47% (11 676/180 528) for those in the highest quartile.

The financial responsibility for paying high annual drug costs OOP was thus distributed very unevenly among these samples of high spenders. In the 1% sample, 37.84% of beneficiaries had actual OOP of <$2000, while others faced much higher liabilities. In the $6560 sample, the fraction of beneficiaries spending less than the cap was even larger, at 64.72%.

Hence, benefits from the cap on OOP are distributed very unevenly, and a substantial minority will see no gain relative to the coverage they had in 2022.

How did those beneficiaries with high spending but low OOP reduce their liability? When we look at the 1% highest spending sample, 84.96% of the beneficiaries in the bottom quartile of OOP costs had other sources to help cover their liabilities. Specifically, these reductions in patient liability payments (PLRO) come from payments made by third-party payers, which lower the amount that beneficiaries are responsible for paying OOP. Notably, only 15.04% of those in the bottom quartile had 0 supplemental assistance from other sources to help reduce their financial liability.

In contrast, if we look at those in the top quartile of OOP liability, only 9.88% had assistance from other sources. Hence, it seems likely that retiree group prescription drug coverage played a major (though variable) role in insulating beneficiaries with high drug spending from high OOP responsibility.

Table 2 illustrates the pattern of external funding of liabilities by OOP quartile. PLRO covered 29.69% of costs for beneficiaries in the lowest OOP quartile, but only 0.13% for those in the highest quartile. Similar trends can be observed in the ratios of outside payments to OOP costs across different OOP quartiles. PLRO spending declines spectacularly across quartiles of OOP spending within our top 1% total spending sample. Mean PLRO payments in the bottom quartiles are almost $30 000 and are on average 84 times larger than OOP payments for these beneficiaries. In contrast, in the top quartiles, mean PLRO payments are <$250 and are on average one fiftieth of OOP payments.

The data specification describes PLRO payments as “the amount of any payment by other third-party payers that reduces the beneficiary's liability for the PDE but does not count toward Part D's true out-of-pocket requirement. Examples include payments by group health plans, worker's compensation, and government programs like the Veterans Administration (VA) and TRICARE.”^7^ Medigap coverage is not permitted to cover drug OOP, and both the VA and Tricare also do not pay for Part D benefits. Hence it is likely that private group insurance with “wrap” coverage that supplements Part D is the primary payor of these additional payments. Government employees and some other private sector workers have such coverage; recent estimates put the percentage of workers with post-retirement drug coverage at about 8%.^8^

## Discussion

The OOP payment cap in Medicare will affect beneficiaries in different ways because the protection offered by their prior coverage varied a great deal. Importantly, in this paper we document a fact that we found surprising; around a third, and possibly more, of high-spending beneficiaries will also be unaffected because they already had coverage from Part D or other sources that limit their financial liability to levels below the cap. While the new provision provides valuable financial and access benefits compared with prior patterns of coverage, they are available to a small and untargeted subset of total beneficiaries.

As a result, there will be an uneven distribution of costs and benefits from the Part D cap. The Congressional Budget Office estimates that the changes in Part D design (including but not limited to the cap) will increase Federal Part D cost by $4 billion in 2031,^9^ offset in part by reductions in medical spending. Those enrollees who previously had private supplements to reduce their liability—about one third of high spenders—will get little of this benefit. However, Medicare does pay a subsidy of 28%^10^ of allowable (equivalent) costs to employers that offer plans for their retirees. The subsidy to such plans is less than that provided to other enrollees. While the incidence of the net cost of post-retirement drug benefits is uncertain (shared partly by employers but largely paid for by reduced income for workers), those agents may continue to make net payments rather than receive full tax-funded assistance unless they change the design of their private benefits.

Because the IRA provisions increase the public subsidy to benchmark Part D coverage, they reduce the value of individual or employer-paid enhanced benefits and other supplements that cover financial liability. Resultingly, there may then be a reduction in the number of people choosing to pay for additional private protection against high OOP payments as they switch to the now more generous Part D benchmark coverage. The resulting patterns of changes in coverage, premiums, and distribution of net benefits will thus be complex and unpredictable. While the number of persons affected will be small, the high cost of coverage per person may lead to noticeable fiscal effects.

## Conclusion

Implementation of the $2000 IRA cap on OOP payments for non-poor Medicare beneficiaries in 2025 will close a major gap in financial protection and access for some of those with high Part D drug expenses. However, a sizeable minority of such persons had already secured other coverage that would limit or cap their financial liability, so the positive benefit from this change will be mitigated. Moreover, if those who previously had other coverage now switch to benchmark Part D plans alone, the additional costs from the subsidies for those plans may lead to an increase in the deficit.

## Supplementary Material

qxaf059_Supplementary_Data

## References

[1] Inflation Reduction Act of 2022 . Public Law 117-169, 136 Stat 1818. 2022.

[2] U.S. Centers for Medicare and Medicaid Services . Inflation Reduction Act and Medicare. CMS.gov. Updated January 20, 2025. Accessed January 23, 2025. https://www.cms.gov/inflation-reduction-act-and-medicare

[3] Office of the Assistant Secretary for Planning and Evaluation, U.S. Department of Health and Human Services . Inflation reduction act research series: Medicare Part D enrollee out of pocket spending: recent trends and projected impacts of the inflation reduction act. July 3, 2023. Updated January 30, 2024. Accessed January 23, 2025. https://aspe.hhs.gov/sites/default/files/documents/1b652899fb99dd7e6e0edebbcc917cc8/aspe-part-d-oop.pdf

[4] Zhou C, Zhang Y. The vast majority of medicare part D beneficiaries still don’t choose the cheapest plans that meet their medication needs. Health Aff (Millwood). 2012;31(10):2259–2265. 10.1377/hlthaff.2012.0087 23048107 PMC3470484

[5] U.S. Centers for Medicare and Medicaid Services . Retiree Insurance & Medicare. Medicare.gov. Accessed January 23, 2025. https://www.medicare.gov/basics/get-started-with-medicare/medicare-basics/working-past-65/retiree-insurance

[6] Abaluck JT, Gruber J. Choice inconsistencies among the elderly: evidence from plan choice in the Medicare Part D program. Working Paper No. 14759. National Bureau of Economic Research. Updated February 2009. Accessed January 23, 2025. http://www.nber.org/papers/w14759

[7] Research Data Assistance Center . Reduction in patient liability due to payment by other payers (PLRO). ResDAC. 2025. Accessed March 8, 2025. https://resdac.org/cms-data/variables/reduction-patient-liability-due-payments-other-payers-plro

[8] Employee Benefit Research Institute . How has the percentage of employers offering retiree health benefits changed? EBRI. Updated February 15, 2024. Accessed February 7, 2025. https://www.ebri.org/health/publications/fast-facts/content/how-has-the-percentage-of-employers-offering-retiree-health-benefits-changed-24

[9] Congressional Budget Office . How CBO estimated the budgetary impact of key prescription drug provisions in the 2022 Reconciliation Act. Updated February 17, 2023. Accessed January 23, 2025. https://www.cbo.gov/publication/58850

[10] U.S. Centers for Medicare and Medicaid Services. Overview of Retiree Drug Subsidy Option. CMS.gov. Updated November 2, 2005. Accessed January 23, 2025 . https://www.cms.gov/medicare/prescription-drug-coverage/employerretireedrugsubsid/downloads/oviewoftherdsrev1.pdf.

